# 908. Little People, Big Data: Eleven-Year Review of 4419 Pediatric Burn Injuries from a Single Center

**DOI:** 10.1093/jbcr/irag033.412

**Published:** 2026-04-06

**Authors:** Morgan M C Karlok, Alexandra R Coward, Felicia N Williams, Lori Chrisco, Booker King, Jamie L Hollowell

**Affiliations:** University of North Carolina, Cary, NC; University of North Carolina, Durham, NC; University of North Carolina, Chapel Hill, NC; University of North Carolina, Durham, NC; University of North Carolina, Durham, NC; North Carolina Jaycee Burn Center, Chapel Hill, NC

## Abstract

**Introduction:**

Despite public health efforts, the incidence and patterns of pediatric burn injuries have remained consistent over the years. Pediatric burn care continues to be delivered across both high- and low-volume centers. This eleven-year review provides a large-volume, single-center analysis with an updated look at current demographics, etiology, acute wound care, and surgical management. Data from high-volume centers are critical to guide discussions on how best to provide specialized, expert-level care to pediatric burn patients. This study examined the demographics, burn characteristics, inpatient course, management strategies, and outcomes in a cohort from one of the nation’s highest-volume pediatric burn centers.

**Methods:**

A retrospective review was conducted on 4419 pediatric patients admitted to and treated at a single ABA-verified pediatric burn center between January 1, 2014, and December 31, 2024. Descriptive statistics were used to analyze patient demographics, injury characteristics, management, and outcomes.

**Results:**

The majority of patients were English-speaking, Caucasian male toddlers aged 1–3 years. Scald burns accounted for 57.5% of cases, most commonly <10% TBSA (91%), with an average TBSA of 3.96%. The mean patient age was 5.08 years. Medicaid was the most common primary insurance (66.1%). ICU admission was required in 315 patients (7.1%), with an average ICU stay of 7.7 days. Inhalation injuries occurred in 22 patients (0.5%), half of whom also had cutaneous burns. Mortality over the study period was 3 patients (0.07%). Nearly half of patients underwent an operation (48.2%), but only 4.0% required more than one procedure. Of those taken to the operating room, 1.9% required autografting, while 6.7% underwent autologous skin cell suspension. Most patients attended at least one follow-up visit (80.9%), resulting in 23 218 pediatric clinic visits during the study period.

**Conclusions:**

This study represents one of the largest single-center pediatric burn datasets with an average of 400 admissions/year. High-volume pediatric burn centers remain rare in the U.S., with most centers treating fewer than 200 pediatric patients per year. Large-scale data such as this provide valuable insights into evolving demographics, management strategies, and outcomes.

**Applicability of Research to Practice:**

Identifying contemporary trends in pediatric burn care is essential for guiding national discussions on best practices and optimizing the organization of care delivery for this population.

**Funding for the study:**

N/A.

